# Effects of preoperative aspirin on perioperative platelet activation and dysfunction in patients undergoing off-pump coronary artery bypass graft surgery: A prospective randomized study

**DOI:** 10.1371/journal.pone.0180466

**Published:** 2017-07-17

**Authors:** Jiwon Lee, Chul-Woo Jung, Yunseok Jeon, Tae Kyong Kim, Youn Joung Cho, Chang-Hoon Koo, Yoon Hyeong Choi, Ki-Bong Kim, Ho Young Hwang, Hang-Rae Kim, Ji-Young Park

**Affiliations:** 1 Department of Anesthesiology and Pain Medicine, Seoul National University College of Medicine, Seoul National University Hospital, Seoul, Korea; 2 Department of Thoracic and Cardiovascular Surgery, Seoul National University College of Medicine, Seoul National University Hospital, Seoul, Korea; 3 Department of Anatomy and Cell Biology, Department of Biomedical Sciences, BK21 Plus Biomedical Science Project, Seoul National University College of Medicine, Seoul, Korea; 4 FACS Core Facility, Seoul National University College of Medicine, Seoul, Korea; Universita degli Studi di Roma La Sapienza, ITALY

## Abstract

The benefit of aspirin use after coronary artery bypass graft surgery has been well proven. However, the effect of preoperative aspirin use in patients undergoing off-pump coronary artery bypass graft surgery (OPCAB) has not been evaluated sufficiently. To evaluate platelet function changes during OPCAB due to preoperative aspirin use, we conducted a randomized controlled trial using flow cytometry and the Multiplate^®^ analyzer. Forty-eight patients scheduled for elective OPCAB were randomized to the aspirin continuation (100 mg/day until operative day) and discontinuation (4 days before the operative day) groups. Platelet function was measured using the platelet activation markers CD62P, CD63, and PAC-1 by flow cytometry, and platelet aggregation was measured using the Multiplate^®^ analyzer, after the induction of anesthesia (baseline), at the end of the operation, and 24 and 48 h postoperatively. Findings of conventional coagulation assays, thromboelastography by ROTEM^®^ assays, and postoperative bleeding—related clinical outcomes were compared between groups. No significant change in CD62P, CD63, or PAC-1 was observed at the end of the operation or 24 or 48 h postoperatively compared with baseline in either group. The area under the curve for arachidonic acid—stimulated platelet aggregation, measured by the Multiplate^®^ analyzer, was significantly smaller in the aspirin continuation group (*P* < 0.01). However, chest tube drainage and intraoperative and postoperative transfusion requirements did not differ between groups. Our study showed that preoperative use of aspirin for OPCAB did not affect perioperative platelet activation, but it impaired platelet aggregation, which did not affect postoperative bleeding, by arachidonic acid.

## Introduction

The use of aspirin after coronary artery bypass graft surgery (CABG) has been proven consistently to be beneficial since the Mangano study [1–4]. However, preoperative use of aspirin has not shown a consistent clinical benefit in patients undergoing CABG [5–7]. Most previous studies of the effect of preoperative aspirin use in this patient population have been retrospective [2–4, 6]. A large randomized clinical trial was conducted recently to examine this issue, but it did not involve the evaluation of platelet function, and aspirin use was randomized on the day of surgery [8]. Moreover, most enrolled patients underwent on-pump CABG. Considering the strong effect of cardiopulmonary bypass (CPB) on the coagulation system, the effect of aspirin use may differ between on-pump CABG and off-pump coronary artery bypass graft surgery (OPCAB).

Flow cytometry is a useful equipment for the assessment of platelet activation. It is a sensitive and powerful method based on laser or impedance analysis. In the flow cytometer, particles are carried to the laser intercept in a fluid stream, enabling the quantification of platelet activation marker expression [9, 10]. The Multiplate^®^ analyzer (Roche Diagnostics, Mannheim, Germany) is a whole-blood impedance aggregometer designed as a point-of-care device for the assessment of platelet function and the effects of antiplatelet agents [11]. It can show the effects of preoperative anticoagulants within a short period of time [12].

In this prospective randomized clinical trial, the effects of preoperative aspirin use on perioperative platelet dysfunction and activation were evaluated using flow cytometry and the Multiplate^®^ analyzer in patients undergoing OPCAB. We hypothesized that preoperative use of aspirin would decrease platelet activation (CD62P expression) and induce platelet dysfunction during perioperative period in these patients.

## Materials and methods

This prospective, randomized, double-blinded, clinical trial was approved by the institutional review board of Seoul National University Hospital, Seoul, Korea (1310-046-526). The study protocol was registered at clinicaltrials.gov (NCT 02209909). Patients were enrolled, after providing written informed consent, between May 2014 and August 2015.

### Patient selection and randomization

Adult (age > 20 years) patients scheduled for elective OPCAB at Seoul National University Hospital were screened for eligibility. Exclusion criteria were coagulation disorder (platelet count < 100,000/microliter, PT INR > 1.2, antithrombin III activity level < 80% or > 120%, fibrinogen level < 2 g/L or > 6 g/L); emergency operation or re-operation; co-existing valvular, liver or kidney disease; cerebral vascular accident <6 months previously, heparin-induced thrombocytopenia; heparin resistance; menstrual phase; preoperative application of CPB or extracorporeal membrane oxygenation device; continuous veno-veno hemofiltration; intra-aortic balloon pump application; myocardial infarction <12 months previously; unstable angina <10 days previously; percutaneous coronary intervention <30 days previously; bare-metal stent insertion <6 weeks previously; drug-eluting stent insertion <12 months previously; and preoperative use of heparin or low-molecular-weight heparin. After enrollment, patients were randomized 1:1 to the aspirin continuation and aspirin discontinuation group. Random sequence of size 2 blocks that included A (letter meaning aspirin continuation group) or B (letter meaning aspirin discontinuation group) were generated and each concealed envelope had one letter within. Enrolled patients were allocated to their groups depending on the letter (A or B) inside the concealed envelopes that were opened up by an anesthesiologist who was unaware of the study.

### Study protocol

Assigned ward nurses delivered drugs to all patients during the perioperative period. In the aspirin continuation group, aspirin (100 mg; aspirin protect^®^, Bayer AG, Leverkusen, Germany) was administered every morning until the operative day. In the aspirin discontinuation group, aspirin was stopped 4 days before the operative day. Other anticoagulants, such as clopidogrel or warfarin were stopped at least 5 days before surgery in all patients.

Anesthetic and surgical techniques were standardized during the trial. A single anesthesiologist and a single surgeon, not aware of the group assignment, performed OPCAB in all patients. All patients arrived in the operating room without premedication. Anesthesia was induced with 0.15 mg/kg midazolam, 1 μg/kg sufentanil and 0.15 mg/kg vecuronium. After endotracheal intubation, mechanical ventilation was applied to maintain an end-tidal carbon dioxide tension of 35–40 mmHg. Anesthesia was provided with target-controlled infusion of propofol (1.5–3.5 ug/ml) and remifentanil (8–20 ng/ml). The Propofol infusion was adjusted to achieve to achieve a bispectral index of 40–60, and the remifentanil infusion was titrated according to the clinical situation. Vecuronium was infused continuously at a rate of 1 μg/kg/min for muscle relaxation. All patients received standard monitoring consisting of five-lead electrocardiography, pulse oximetry, invasive radial artery pressure measurement, pulmonary artery pressure measurement, nasopharyngeal temperature measurement, and transesophageal echocardiography.

All the patients underwent OPCAB involving an internal thoracic artery or a saphenous vein graft after systemic heparinization to maintain an activated clotting time > 300s. During distal anastomoses, intracoronary shunts (Axius^™^; Guidant, Cupertino, CA, USA) or intraluminal occluders (Flo-Rester^®^; Synovis Surgical Innovations, St Paul, MN, USA) were used according to the grade of coronary stenosis or coronary artery territories. After anastomoses had been completed, heparin effects were reversed with protamine sulfate. The cardiac index was maintained at > 2.0 L/min/m^2^ using echocardiographic guidance and cardiac index monitoring with a Swan-Ganz catheter (Edwards Lifesciences, Irvine, CA, USA). After the completion of surgery, patient-controlled analgesia with intravenous morphine or oxycodone was provided for pain control. In all patients of a dual antiplatelet agent combining 100mg of aspirin and 75mg of clopidogrel was started according to the clinical process, between 24 and 48 h postoperatively. All patients underwent early follow-up coronary angiograms on postoperative day 1. Blood samples were collected for the analysis of CD62P, CD63, PAC-1, and aggregation; determination of the coagulation profile and performance of ROTEM^®^ assay at the following time points: immediately after anesthesia induction (T1), at the end of the operation (T2), 24 h postoperatively (T3), and 48 h postoperative (T4). Troponin I was also evaluated at determined time points.

### Clinical assessments

The primary study endpoint was the increment of CD62P expression at 48 h postoperatively. CD62P expression was measured by flow cytometry [13, 14]. The secondary endpoints were the area under curve (AUC) of platelet aggregation, measured with the Multiplate^®^ analyzer and postoperative chest tube drainage. Preoperative, intraoperative variables and postoperative clinical outcomes including death, stroke, thrombotic occlusion, and intensive care unit (ICU) stay, troponin I were also recorded.

### Coagulation and platelet activity assays

#### Flow cytometry

Blood was drawn into 5-mL round-bottomed polystyrene tubes (Becton Dickinson, Franklin Lakes, NJ, USA). After centrifugation at 600 rpm for 20 min, platelet-rich plasma (PRP) was removed and placed in clear 15-mL conical tubes. An equal volume of 2% formaldehyde was added and mixed. The mixed samples were kept at room temperature for 10 min. Then, 10 mL washing solution (PBS + 1% FBS) was added. The PRP was prepared by centrifugation at 2000 rpm for 10 min. Fixed PRP samples were kept stored at 4°C for up to 8 h. Saturating concentrations of antibodies against P-selectin (APC-conjugated anti-CD62P, AK-4 clone; Beckton Dickinson), an activated version of the gpIIb/IIIa receptor (FITC-conjugated anti-PAC-1, PAC-1 clone, Beckton Dickinson) and CD63 (V450-conjugated anti-CD63, H5C6 clone; Beckton Dickinson) were then added. After 30 min incubation at room temperature in the dart, centrifugation was performed at 2000 rpm for 5 min.

The samples were analyzed within 12 h in a FACSAria III cell-sorter flow cytometer (Becton Dickinson) equipped with four lasers and FACSDiva^™^ software (Beckton Dickinson Immunocytometry Systems, Franklin Lakes, NJ, USA).

An unstained sample and uniform microspheres (0.49-μm diameter; Bangs Laboratories, Inc., Fishers, IN, USA) were used to adjust the FS/SS PMT voltages. Gating platelets and single fluorochrome histogram plots were used to adjust FL voltages to a sensitivity level at which all unstained platelets were negative (<10^1^ log). Light scatter and fluorescence data from 10,000 platelet events were collected with all detectors in logarithmic mode. The platelet population was identified by its light-scattering characteristics. A singly or dually qualified specialist in biochemistry and/or anatomy. Conducted chemical procedures on all samples.

#### Platelet aggregation tests

Platelet aggregation studies were performed using the aggregation agonists adenosine diphosphate (ADP; 6.4 μmol/L), and arachidonic acid (ASPI; 0.5 μmol/L) with the Multiplate^®^ analyzer. Results showed maximum platelet aggregation, expressed as the AUC over 6 min.

#### Conventional coagulation assay

Complete blood count, D-dimer tests, and measurement of prothrombin time (PT), activated partial prothrombin time (aPTT), and fibrinogen concentration were performed using whole blood. The whole blood sample was transferred to laboratory and all of above tests were performed there.

#### Thromboelastography

EXTEM, INTEM, FIBTEM and HEPTEM analyses were performed according to standard techniques using a ROTEM^*®*^ device (Pentapharm GmbH, Munich, Germany) [15]. The Main parameters documented were clotting time (CT), clot formation time (CFT), amplitude of clot firmness 10 min after CT (A10) and maximum clot firmness (MCF).

### Statistical analyses

Bednar et al. presented 53% increment of CD62P expression at postoperative 48 hours in patients who stopped administration of preoperative aspirin in OPCAB [16]. We used these data to find out sample size for our study. Assuming a statistical power of 0.8 and a type 1 error of 0.05, we calculated that a sample of 24 patients per group was needed to test the hypothesis that the aspirin continuation group would show a clinically significant (30%) decrease in the postoperative CD62P increment compared with the aspirin discontinuation group assuming standard deviation of 19% in each group.

Linear mixed-effects models for analysis of repeated measures were performed to compare the increments of CD62P, CD63, and PAC-1 between groups and to compare aggregation, conventional coagulation assay and ROTEM^®^ assay results between groups. The mixed linear models included random intercept for each subject and fixed effects for group, time and the interaction between group and time. Poisson regression was used for platelet count using the generalized estimating equation with interchangeable covariance matrix. The significance of interaction term between group and time was tested and non-significant interaction was excluded in the final model. Normality assumption of residuals for each linear model was checked by histograms and normal quantile-quantile plots of residuals. The plots showed no violation of normality assumption. When there was significant time effect, P-value of less than .017 were considered statistically significant using Bonferroni correction for 3 comparisons of times (T2, T3, T4) compared with T1. The independent t test, Fisher’s exact test and Mann-Whitney test were used to compare patient characteristics and surgery-related variables according to the normality of data distribution. Gender and past medical history were compared between groups using Fisher’s exact test. Data were presented as mean ± Standard deviation, median [interquartile range], and number of patients (%). All statistical analyses were performed with IBM SPSS Statistics software (version 21, SPSS Inc., IBM Corporation, USA).

P-values of less than .017 were considered statistically significant using Bonferroni correction for 3 comparisons in each outcome.

## Results

**Fig 1:**
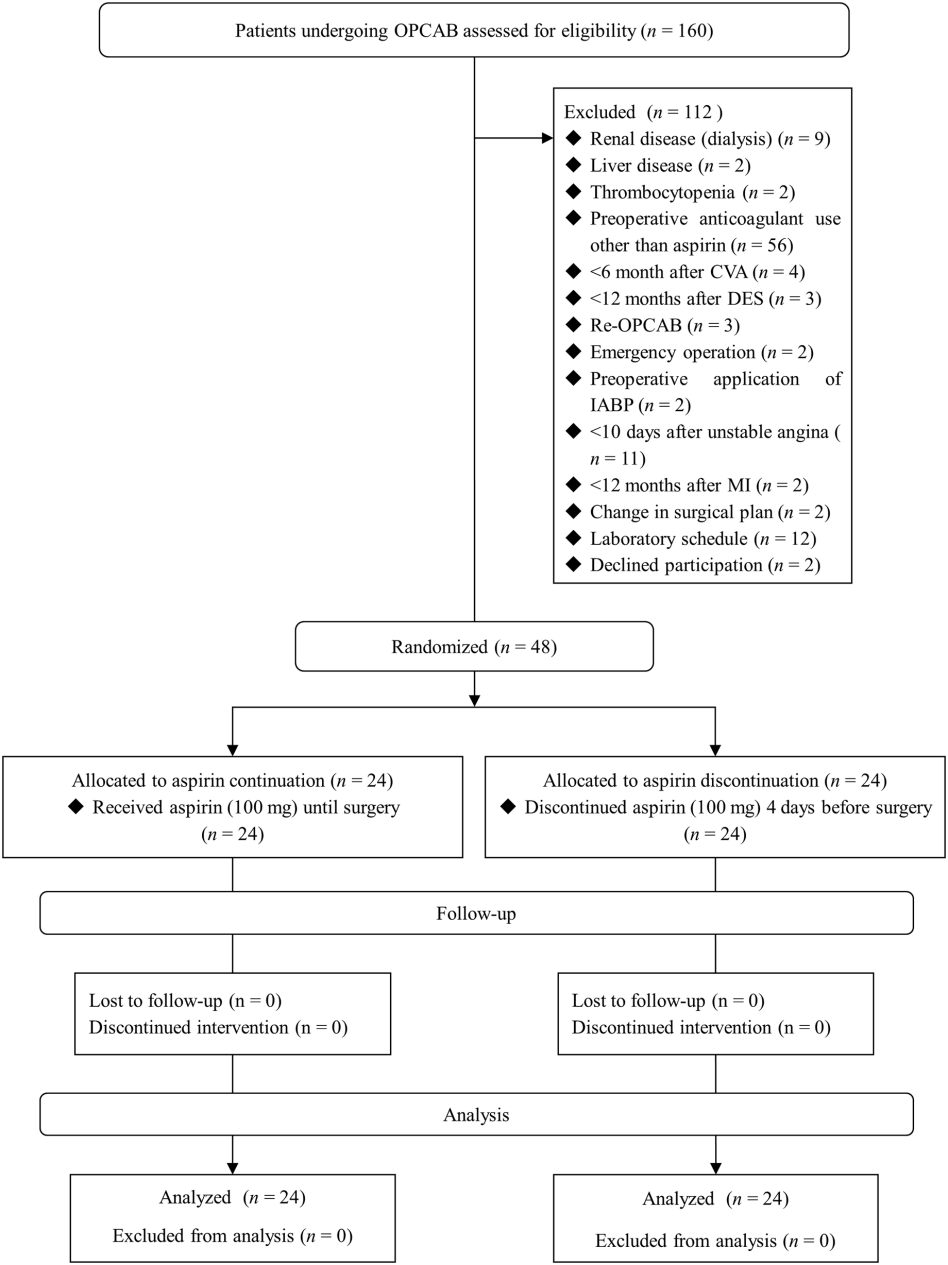
CONSORT flow diagram.

Between May 2014 and August 2015, 160 patients received elective OPCAB and 112 patients were excluded from this study. Forty-eight patients were randomized and included in the final analyses (Fig 1). Preoperative patient characteristics and variables related to anesthesia and surgery did not differ between groups (Tables 1 and 2).

**Table 1:** Patient characteristics.

	Aspirin continuation (n = 24)	Aspirin discontinuation (n = 24)	*P*
Sex (M/F)	19/5	18/6	>0.99
Age (years)	66 ± 8	67 ± 12	0.70
Height (cm)	165 ± 7	162 ± 8	0.24
Weight (kg)	63 ± 11	66 ± 11	0.43
Body mass index (kg/m^2^)	23.2 ± 2.9	25.0 ± 3.8	0.07
Past medical history			
Myocardiac infarction	1 (4.2)	5 (20.8)	0.19
Hypertension	15 (62.5)	16 (66.7)	>0.99
Diabetes mellitus	17 (70.8)	11 (45.8)	0.14
Cerebrovascular accident	0 (0)	4 (16.7)	0.11
Hyperlipidemia	5 (20.8)	7 (29.2)	0.74
Smoking	5 (20.8)	8 (33.3)	0.52
Ejection fraction (%)	59 [55–62]	56 [53–61]	0.24
EuroSCORE	4.0 ± 2.6	4.3 ± 2.6	0.75
Duration of previous aspirin use			0.78
no medication	4	5	
<1 month	2	4	
1–12 months	4	3	
>12 months	14	12	

Values are expressed as *n*, mean ± SD, median (interquartile range) or number of patients (%). Independent t-test, Fisher’s exact test and Mann-Whitney test were used to compare groups. EuroSCORE, European System for Cardiac Operative Risk Evaluation.

**Table 2:** Intraoperative and postoperative variables.

	Aspirin continuation (n = 24)	Aspirin discontinuation (n = 24)	*P*
Number of grafts	4 [3–4]	4 [3–4]	0.83
Dose of intraoperative heparin (mg)	154 [140–180]	156 [138–181]	0.84
Dose of intraoperative protamine (mg)	84 [70–100]	71 [64–97]	0.07
Transfused blood components			
Packed red blood cells (units)	1 [0.75–3]	1 [0.75–2]	0.96
Fresh frozen plasma (units)	0	0	-
Platelet concentrate (units)	0	0	-
Autologous whole blood (mL)	250 [100–500]	125 [100–355]	0.26
Duration of anesthesia (min)	454 ± 60	452 ± 85	0.93
Duration of surgery (min)	378 ± 56	373 ± 79	0.85
Chest tube drainage (mL)	919 [763–1333]	811 [667–1049]	0.21
Packed red blood cell in ICU (units)	0.5 [0–2]	0 [0–1]	0.21
Fresh frozen plasma in ICU (units)	0	0	-

Values are expressed as *n*, mean ± SD or median [interquartile range]. The independent *t* test and Mann-Whitney test were used to compare groups. ICU, intensive care unit.

### Flow cytometry findings

**Fig 2:**
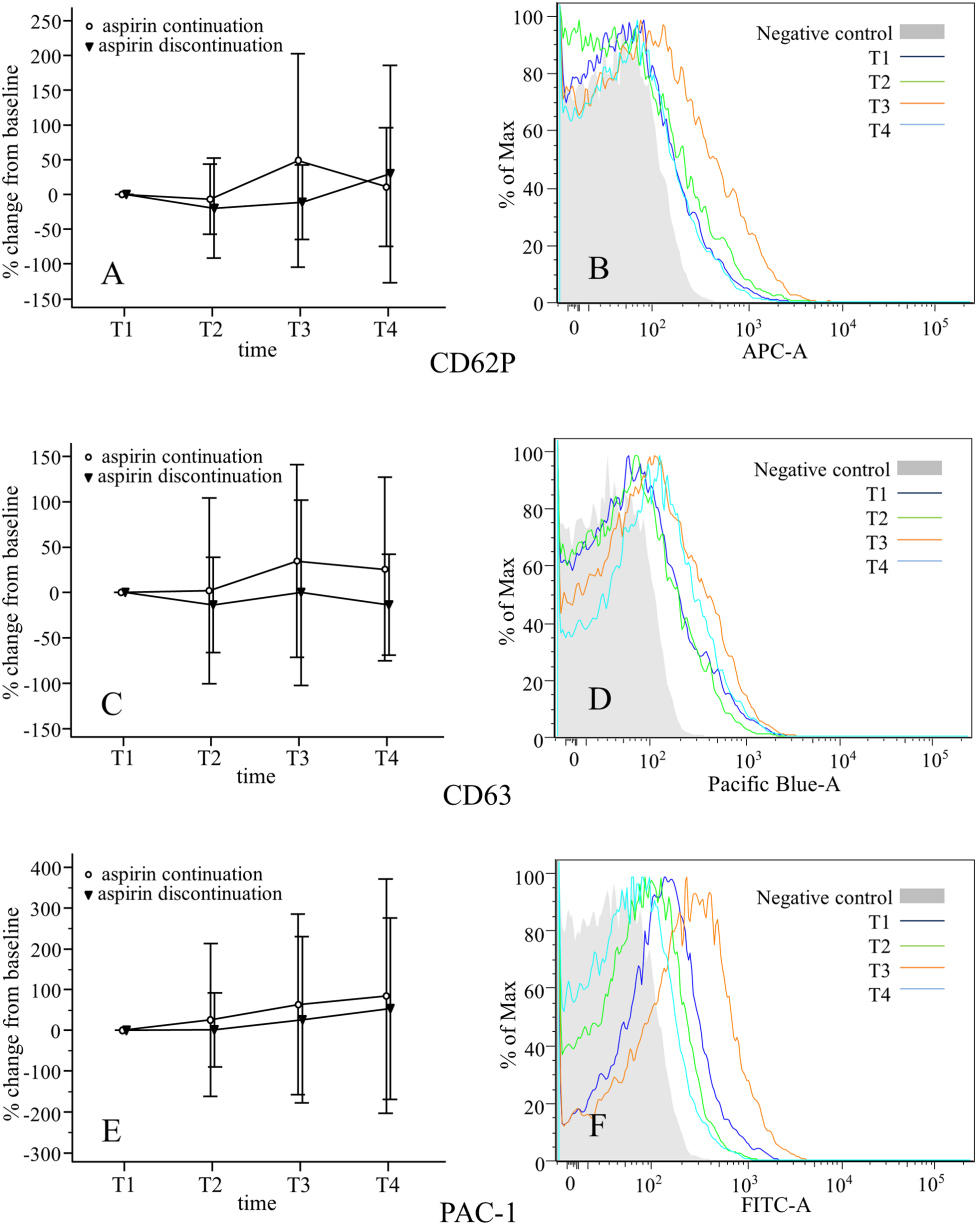
Increments of surface expression of P-selectin (CD62P; A, B), glycoprotein 53 (CD63; C, D), and activated GP IIb/IIIa (PAC-1; E, F) on platelets from patients undergoing OPCAB. Data are expressed as means with standard deviations (A, C, E), representative flow cytometry histogram (B, D, F).

Compared with baseline, the changes of expressions of platelet antigen CD62P, CD63, and PAC-1 did not differ T2, T3, or T4 in either group. There was no significant difference of increments of CD62P, CD63, and PAC-1 at the end of operation and 24/48 h after operation compared to baseline values between two groups (Fig 2, S1 Table).

### Platelet aggregation

**Fig 3:**
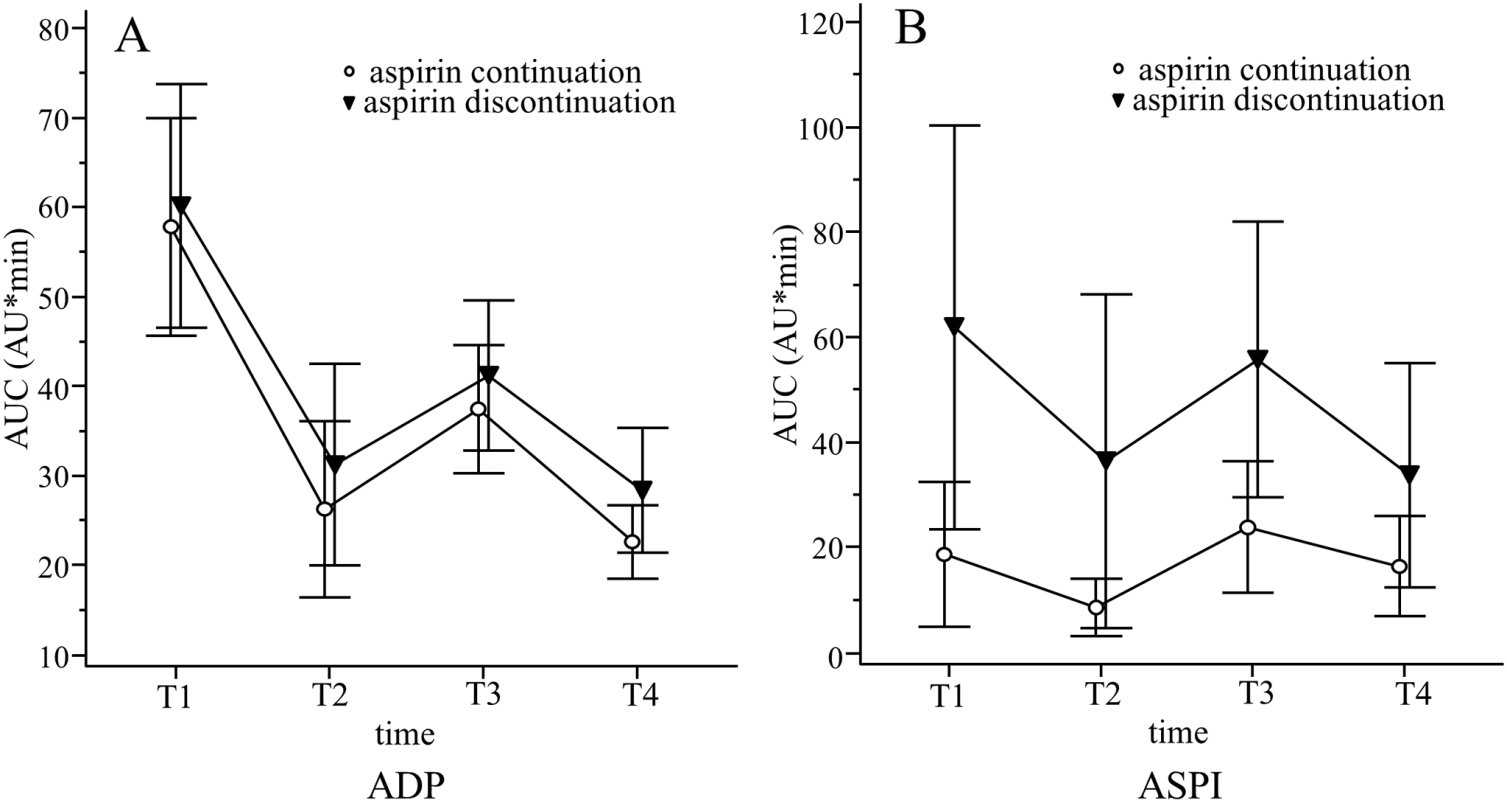
Platelet aggregation induced by ADP and arachidonic acid, as determined with the Multiplate^®^ analyzer, in patients undergoing OPCAB. T1, immediately after induction; T2, at the end of the operation; T3, 24 h postoperatively; T4, 48 h postoperatively. Data were analyzed using mixed-effect models for repeated measures with Bonferroni adjustment. Data are expressed as means with standard deviations. **A. ADP-induced platelet aggregation**. *P < 0.05, significant time effect (vs. T1) in both groups. **B. Arachidonic acid-induced platelet aggregation**. **P < 0.05, significant interaction between group and time at all timepoints in both groups. † *P* < 0.05 between groups at all timepoints.

ADP mediated platelet aggregation differed significantly over time (*P* < 0.01), but not between groups (Fig 3A). ASPI-induced platelet aggregation differed between groups and over time (both *P* < 0.01). The AUC was significantly greater for the aspirin discontinuation group than for the aspirin continuation group at T1-T4 (all *P* < 0.01; Fig 3B).

### Conventional coagulation assay

Among conventional coagulation assay results, only the platelet count differed between groups; it was significantly higher in the aspirin discontinuation group at T1. Mean platelet count in aspirin discontinuation group was 1.217 times higher than that in aspirin continuation group (*P* = 0.002, RR, 95% CI = 1.217, 1.074–1.380; Tables 3 and 4). There was no difference in the other parameters between the two groups (*P* > 0.05). The PT was prolonged at T2, T3 and T4 relative to T1 in both groups (all *P* < 0.01). Fibrinogen levels were significantly decreased at T2 relative to T1 in both groups (*P* < 0.01). The D-dimer level was increased at T3 relative to T1 in both groups (*P* < 0.01).

**Table 3:** Conventional coagulation assay results.

		T1	T2	T3	T4
Hb (g/dL)	Aspirin continuation	12.3 ± 1.7 (24)	11.7 ± 1.3 (24)	11.6 ± 1.1 (23)	11.0 ± 1.1 (23)
	Aspirin discontinuation	12.1 ± 1.3 (24)	12.2 ± 1.2 (24)	12.2 ± 0.8 (24)	11.5 ± 1.3 (24)
Platelet count (k/μL)	Aspirin continuation	187 ± 34 (24)	129 ± 34 (24)	136 ± 32 (23)	126 ± 31 (23)
	Aspirin discontinuation	228 ± 61 (24)	152 ± 55 (24)	154 ± 60 (24)	134 ± 60 (23)
PT (INR)	Aspirin continuation	1.06 ± 0.08 (24)	1.19 ± 0.12 (24)	1.26 ± 0.17 (23)	1.18 ± 0.14 (15)
	Aspirin discontinuation	1.08 ± 0.06 (24)	1.18 ± 0.09 (24)	1.27 ± 0.16 (24)	1.20 ± 0.13 (18)
aPTT (s)	Aspirin continuation	31.8 ± 5.2 (24)	32.4 ± 4.9 (24)	33.2 ± 6.5 (23)	35.3 ± 8.7 (15)
	Aspirin discontinuation	33.9 ± 6.7 (24)	35.0 ± 6.9 (24)	37.3 ± 14.5 (24)	37.7 ± 11.9 (17)
Fibrinogen (mg/dL)	Aspirin continuation	332 ± 62 (24)	242 ± 83 (24)	348 ± 72 (23)	491 ± 84 (15)
	Aspirin discontinuation	325 ± 62 (24)	246 ± 63 (24)	366 ± 81 (24)	532 ± 90 (15)
D-dimer (μg/mL, FEU)	Aspirin continuation	0.81 ± 1.09 (22)	1.05 ± 0.95 (22)	1.64 ± 1.21 (19)	1.28 ± 0.64 (19)
	Aspirin discontinuation	0.54 ± 0.50 (24)	0.84 ± 0.53 (24)	1.49 ± 0.83 (21)	1.64 ± 1.78 (21)

Values are expressed as mean ± SD, (n). Data were obtained by comparison of linear mixed-effect models with repeated measures using datasets with four data points. T1, after anesthesia induction; T2, at the end of the operation; T3, 24 h postoperatively; T4, 48 h postoperatively; Hb, hemoglobin; PT, prothrombin time; aPTT, activated partial thromboplastin time; FEU, fibrinogen equivalent units.

**Table 4:** Linear mixed effects and Poisson regression models for conventional coagulation assay.

			**Estimate (Mean)**	**SE**	**P-value**	**P-value (Overall time effect)**
**Linear mixed-effects model**						
Hb (g/dL)						
Fixed effects						
		Intercept	12.033	0.223	<.0001	
	Group	Continuation				
		Discontinuation	0.343	0.263	0.1986	
	Time	T1				<.0001
		T2	-0.248	0.201	0.2201	
		T3	-0.297	0.203	0.1451	
		T4	-0.942	0.203	<.0001	
Random effects						
Variance random intercepts			0.584	0.175	0.0008	
Residual variance			0.972	0.117	<.0001	
PT (INR)						
Fixed effects						
		Intercept	1.067	0.023	<.0001	
	Group	Continuation				
		Discontinuation	0.007	0.030	0.8046	
	Time	T1				<.0001
		T2	0.115	0.016	<.0001	
		T3	0.197	0.016	<.0001	
		T4	0.115	0.018	<.0001	
Random effects						
Variance random intercepts			0.009	0.002	<.0001	
Residual variance			0.006	0.001	<.0001	
aPTT (s)						
Fixed effects						
		Intercept	31.444	1.533	<.0001	
	Group	Continuation				
		Discontinuation	2.761	1.785	0.1287	
	Time	T1				<.0001
		T2	0.873	1.423	0.5404	
		T3	2.551	1.432	0.0773	
		T4	3.437	1.635	0.0376	
Random effects						
Variance random intercepts			24.647	7.937	0.0019	
Residual variance			47.923	6.085	<.0001	
Fibrinogen (mg/dL)						
Fixed effects						
		Intercept	322.030	14.409	<.0001	
	Group	Continuation				
		Discontinuation	12.853	18.031	0.4795	
	Time	T1				<.0001
		T2	-84.313	11.210	<.0001	
		T3	27.880	11.290	0.0149	
		T4	170.200	13.151	<.0001	
Random effects						
Variance random intercepts			3047.920	811.340	0.0002	
Residual variance			3016.170	384.660	<.0001	
D-dimer (μg/mL, FEU)						
Fixed effects						
		Intercept	0.699	0.183	0.0004	
	Group	Continuation				
		Discontinuation	-0.073	0.212	0.7336	
	Time	T1				<.0001
		T2	0.264	0.170	0.1226	
		T3	0.881	0.177	<.0001	
		T4	0.807	0.177	<.0001	
Random effects						
Variance random intercepts			0.353	0.113	0.0018	
Residual variance			0.657	0.084	<.0001	
**Poisson regression model with GEE**			**RR**	**SE**	**P-value**	**P-value (Time effect)**
Platelet count (k/μL)						
		Intercept	187.017	1.037	<.0001	
	Group	Continuation				
		Discontinuation	1.217	1.066	0.0022	
	Time	T1				<.0001
		T2	0.677	1.037	<.0001	
		T3	0.696	1.037	<.0001	
		T4	0.627	1.042	<.0001	

RR, Relative risk

### Thromboelastographic findings

ROTEM^®^ parameters on the EXTEM, INTEM, FIBTEM, and HEPTEM tests did not differ between groups (S2 Table).

### Postoperative bleeding and other clinical outcomes

Chest tube drainage, intraoperative transfusion, postoperative transfusion requirements did not differ between two groups (Table 2). Hemoglobin levels were similar in the two groups at all time points (Table 3). Troponin-I increased significantly at T2 and T3 compared with T1in both groups (S1 Fig).

No conversion to on-pump CABG occurred in either group. Re-operative management of thrombotic occlusion of a saphenous vein graft was required in one patient in the aspirin continuation group and two patients in aspirin in the aspirin discontinuation group. No re-operation was performed due to bleeding in either groups. Two patients in the aspirin continuation group had embolic strokes, and one patient died of this cause. Another patient in the aspirin continuation group died due to postoperative metabolic acidosis and multi-organ failure. ICU stay did not differ between the aspirin continuation and discontinuation groups (44 h [27–51] *vs*. 45 h [29–69]). There was no difference in the Postoperative hospital stay between two groups (10 d [8–12] *vs*.10 d [8–17]).

## Discussion

In this study of 48 patients undergoing OPCAB, platelet activation measured by increment of CD62P, CD63, and PAC-1 did not differ between the aspirin continuation and discontinuation groups until postoperative day 2. The AUC obtained by the ASPI test was significantly lower in the aspirin continuation group than in the aspirin discontinuation group during the perioperative period. However, chest tube drainage and perioperative transfusion requirements were similar in the two groups.

Using flow cytometry, Bednar et al. [16] detected a 53% increment of CD62P expression at 48 h postoperative in patients who underwent OPCAB after preoperative aspirin discontinuation, compared with those who underwent on-pump CABG after preoperative aspirin discontinuation. This findings suggests that platelet activation during the early postoperative period could be more serious during OPCAB than during on-pump CABG. However, the results of the current study show that preoperative aspirin use dose not effectively reduce this platelet activation during OPCAB.

There are multiple pathways in platelet activation [17–23]. Also, Thrombin, ADP, and thromboxane A_2_ are released by activated various cells and they trigger platelet activation through G protein-coupled receptors [24]. Among those triggering factors, thrombin is powerful factor in platelet activation through protease-activated receptors [17, 18, 22, 23]. It is well known that activated platelets produce thromboxane A_2_ through cyclooxygenase. As well as it plays a key role in platelet aggregation, it activates surrounding platelets after degranulation [25]. Moreover, a previous study presented that thromboxane A_2_ amplified the response to other potent platelet agonists [26]. However, platelet activation did not differ between groups regardless of aspirin pretreatment in our study. This result suggested that the potency of thromboxane A_2_ on platelet activation may be smaller than other mechanisms because aspirin inhibit production of thromboxane A_2_.

Results of a recent retrospective study involving 3018 patients suggest that low-dose aspirin use within 24 h of CABG with CPB decreased early postoperative mortality [2]. Another retrospective study involving 1418 patients showed that preoperative aspirin use increased angina recurrence-free survival rates after OPCAB slightly [3]. The authors suggested that preoperative aspirin use protected against postoperative thrombotic complication. However, both of these studies are limited by their retrospective nature. In a prospective observational clinical study, Suwalski et al. [27] investigated the effect of preoperative aspirin use with a myocardial enzyme and platelet function analyzer (PFA-100^®^; Dade Behring, Germany). Their findings suggested that preoperative aspirin use did not protect against acute platelet activation in OPCAB. A recent large clinical trial examining CABG [8], and non-cardiac surgery [28] showed that preoperative aspirin use did not prevent postoperative thrombotic complication. These prospective study results are consistent with those of the current study, in which preoperative aspirin use did not affect perioperative platelet activation and postoperative thrombotic complication.

Recently, Myles et al. [8] showed that the administration of preoperative aspirin did not increase postoperative bleeding compared with placebo in patients undergoing CABG. Our results also supported their results. However, in our study, platelet aggregation by ASPI was attenuated in aspirin continuation group even if that did not result in any difference of postoperative bleeding between two groups. This might be interesting as it is also known that aspirin ironically attenuates its antithrombotic effect by enhancing production of isoprostanes that favoring platelet recruitment [29–32].

Numerous studies have investigated aspirin resistance since Helgason et al. introduced its concept in 1994 [33, 34]. There is a possibility that preoperative aspirin resistance may affect the result of our study [35, 36]. In such an aspect, the negative result on platelet activation might be partly caused by aspirin resistance. However, we hoped that our result would be pragmatic and our study result may reflect that the usual patents instead of aspirin responders, only. So, we did not exclude the aspirin non responders in our study.

In our results, ADP-induced aggregation changed over time, but there was no differences in the trend of changes between groups. On the contrary, ASPI-induced aggregation changed both over time, and between groups. ADP-induced aggregation and ASPI-induced aggregation are used to evaluate platelet dysfunction by P2Y12 platelet inhibitor and aspirin, respectively. And, their effect on inhibition of platelet activations are mediated by different pathway. Different platelet aggregation patterns between groups in ASPI-induced aggregation could mean different pathways inhibiting platelet activation. To evaluate further mechanism, further analysis may be necessary such as serum thromboxane level which is related in ASPI-induced aggregation.

We did not investigate oxidative stress such as the oxLDL and H_2_O_2_ level in this study, which are well known to induce platelet activation, especially in promoting platelet aggregation. Many studies attempted to investigate the effect of aspirin on vascular oxidative stress [37–40]. Most of them presented that aspirin protects endothelial cells from oxidative stress or reverse the impairment of endothelium [38–40]. Also, one previous study presented that aspirin reduce vascular oxidative stress by inhibiting the expression of the LOX-1 receptor that is induced by oxidized low density lipoprotein (oxLDL) in endothelial cells [41]. Our study focused the effect of preoperative aspirin use on platelet activity. If we checked the plasma level of oxidative stress such as oxLDL or H_2_O_2_, it may be possible to investigate whether aspirin influence the oxidative stress level.

We expected that OPCAB would be similar to major vascular surgery, rather than cardiac surgery, in terms of hemostasis (or coagulation) and the non-use of CPB. In addition, the strategy used to maintain coagulation status in OPCAB is usually similar to that used in non-cardiac surgery [42, 43]. Devereaux et al. [28] reported that preoperative aspirin administration did not reduce mortality and caused major bleeding only in non-cardiac surgery. This result may be consistent with the perioperative impaired platelet aggregation observed in the current study. However, no increased bleeding occurred in aspirin continuation group in our study like as previous some studies.

In our study, one patient in the aspirin continuation group and two patients in aspirin discontinuation group required re-operation due to saphenous graft failure in the early postoperative period. In the study conducted by Poston et al. [44], OPCAB did not significantly increase platelet activation, as measured by thromboelastography and whole-blood aggregometry. Moreover, they showed that platelet function did not differ between patients who developed graft thrombosis and those with patent grafts. In our study, platelet aggregation was lower in aspirin continuation group. However, thrombotic complications were not statistically different between two groups in our study population. Therefore, whether ASPI-induced attenuation of platelet aggregation in the perioperative period is beneficial for graft patency seems not to be clear yet.

This study has several limitations. First, many patients were excluded because they received different anticoagulation therapies, such as low-molecular-weight heparin, heparin, and clopidogrel (Fig 1). As various anticoagulation management strategies are applied to the patients undergoing CABG, our results may not be applied to the patients undergoing OPCAB cases. Second, the primary endpoint was platelet activation, measured by flow cytometry, and the patient sample was small. Further studies with larger samples are needed to evaluate the effects of attenuated perioperative platelet aggregation on clinical outcomes such as postoperative bleeding and graft patency. Finally, we stopped aspirin 4 days before the surgery, which might not be sufficient to distinguish an aspirin discontinuation effect. Aspirin discontinuation 5–7 days before surgery can increase the risk of the thromboembolic event by the progressive recovery in platelet activity as “rebound phenomenon” [45, 46]. We expected that aspirin cessation for <5 days would have a minimal effect on thromboembolic risk in patients with coronary artery disease.

In conclusion, preoperative aspirin use did not affect platelet activation in the early postoperative period in patients undergoing OPCAB. However, it decreased ASPI-induced platelet aggregation. Preoperative aspirin may not prevent postoperative complications by platelet activation, but may increase the possibility of bleeding by platelet dysfuction. Further studies of the preoperative use of aspirin and bleeding tendency in patients undergoing OPCAB are needed.

## Supporting information

S1 Fig Perioperative changes in troponin I. Results are shown as mean ± SD. Data were analyzed with a linear mixed-effects model for repeated measures with Bonferroni adjustment. Perioperative TnI level did not differ between groups at any timepoint.
*, †; The TnI increased significantly at T2 and T3 relative to T1 in both groups (both *P* < 0.01).
TnI, troponin I; T1, immediately after induction; T2, at the end of the operation; T3, 24 h postoperatively; T4, 48 h postoperatively.
(TIF)

S2 Fig Histogram of studentized residuals. The histogram plots of studentized residuals seemed to be roughly normally distributed and the normality assumption of residuals did not seem to be seriously violated in the plots. We used a mixed effects model for analysis. In addition, according to the simulation study of Jacqmin-Gadda et al, linear mixed model is relatively robust to deviations from normality [47].
(TIF)

S1 Table Platelet activation markers results. (DOCX)

S2 Table ROTEM^®^ profile. Results are shown as mean ± SD. Data were analyzed using a linear mixed-effect model for repeated measures with Bonferroni adjustment. No significant difference was observed between groups.
CT, Clotting time; A10, amplitude of clot firmness 10 min after CT; CFT, clot formation time; MCF, maximum clot firmness.
(DOCX)

S1 File CONSORT checklist. (DOCX)

S2 File Clinical research protocol (Original language version). (DOCX)

S3 File Clinical research protocol (English language version). (DOCX)

S4 File Certification of approval (English language version). (PDF)

S5 File A dataset for the present study. (XLSX)
