# Supplementary figures and images for: Effects of preoperative aspirin on perioperative platelet activation and dysfunction in patients undergoing off-pump coronary artery bypass graft surgery: A prospective randomized study
Source: PLoS One. 2017 Jul 17;12(7):e0180466. doi: 10.1371/journal.pone.0180466 (PMC5513419; doi:10.1371/journal.pone.0180466)

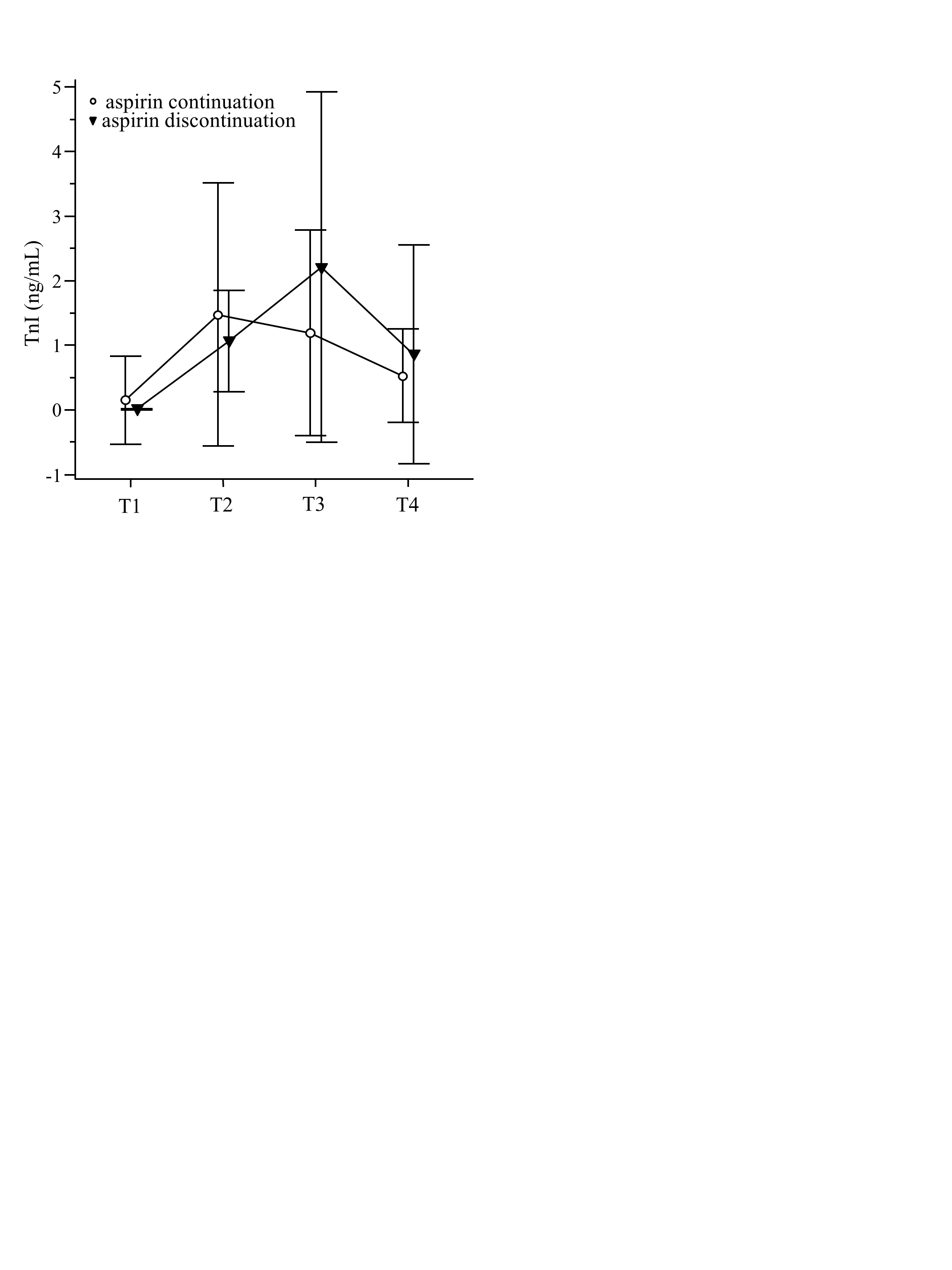

Supplement: S1 Fig — Results are shown as mean ± SD. Data were analyzed with a linear mixed-effects model for repeated measures with Bonferroni adjustment. Perioperative TnI level did not differ between groups at any timepoint. *, †; The TnI increased significantly at T2 and T3 relative to T1 in both groups (both P < 0.01). TnI, troponin I; T1, immediately after induction; T2, at the end of the operation; T3, 24 h postoperatively; T4, 48 h postoperatively. (TIF) [file pone.0180466.s001.tif]

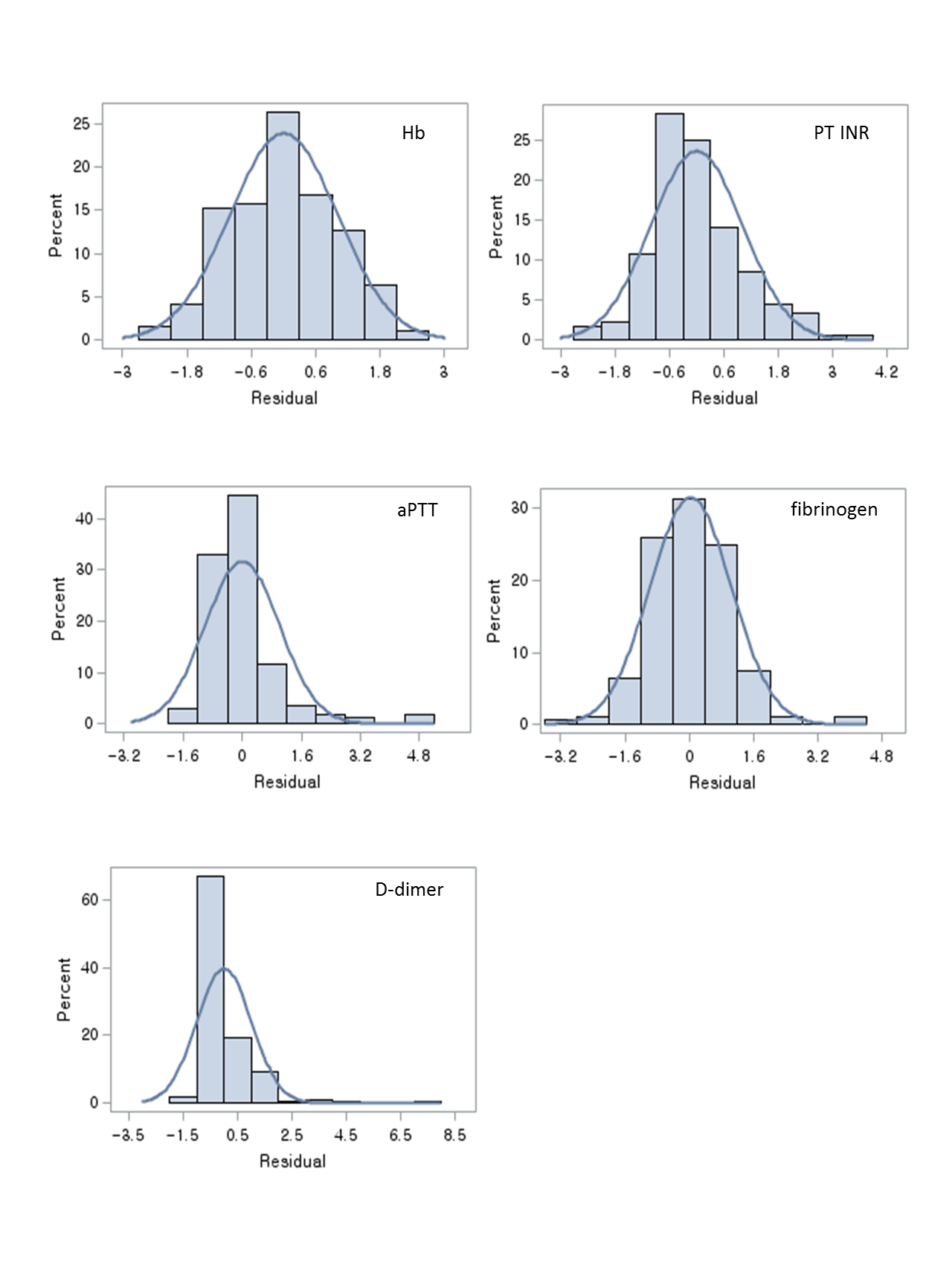

Supplement: S2 Fig — The histogram plots of studentized residuals seemed to be roughly normally distributed and the normality assumption of residuals did not seem to be seriously violated in the plots. We used a mixed effects model for analysis. In addition, according to the simulation study of Jacqmin-Gadda et al, linear mixed model is relatively robust to deviations from normality [47]. (TIF) [file pone.0180466.s002.tif]
