# Supplementary material for: Effects of preoperative aspirin on perioperative platelet activation and dysfunction in patients undergoing off-pump coronary artery bypass graft surgery: A prospective randomized study
Source: PLoS One. 2017 Jul 17;12(7):e0180466. doi: 10.1371/journal.pone.0180466 (PMC5513419; doi:10.1371/journal.pone.0180466)
Supplement: S1 Table — (DOCX) [file pone.0180466.s003.docx]

**S1. Platelet activation markers results**

|  |  | T1 | T2 | T3 | T4 |
| --- | --- | --- | --- | --- | --- |
| Increment of CD62P (%) | Aspirin continuation | 0 | -7.1± 50.6 | 48.9 ± 153.8 | 10.6± 85.6 |
|  |  | 0 | -14.7  [-82.4 ,121.7] | -25.4  [-87.5, 456.0] | -28.8  [-89.3, 212.1] |
|  | Aspirin discontinuation | 0 | -21.0± 70.2 | -10.5± 51.6 | 36.3± 150.8 |
|  |  | 0 | -42.0  [-87.0 ,172.4] | -30.6  [-74.2, 456.0] | -25.0  [-97.5, 533.3] |
| Increment of CD63 (%) | Aspirin continuation | 0 | 1.6± 102.1 | 34.7± 106.5 | 25.9± 101.3 |
|  |  | 0 | -33.0  [-82.5, 404.8 ] | 1.0  [-72.0, 383.3 ] | 0  [-88.5, 363.8 ] |
|  | Aspirin discontinuation | 0 | -17.7± 51.8 | -3.2± 98.5 | -11.4± 53.2 |
|  |  | 0 | -35.3  [-80.4, 126.1 ] | -39.2  [-73.8, 371.4 ] | -21.1  [-89.2, 159.6 ] |
| Increment of PAC-1 (%) | Aspirin continuation | 0 | 25.1± 187.7 | 63.6± 222.2 | 83.9± 287.0 |
|  |  | 0 | -29.8  [-99.1, 842.1] | -2.1  [-96.7, 994.7] | -19.9  [-96.7, 1010.5] |
|  | Aspirin discontinuation | 0 | 111.6± 547.6 | 99.1± 431.3 | 444.6± 1835.2 |
|  |  | 0 | -18.3  [-100.0, 2650.0] | -33.4  [-100.0, 1900.0] | 0  [-100.0, 8400.0] |

Values are expressed as mean ± SD, median [min, max].

T1, after anesthesia induction; T2, at the end of the operation; T3, 24 h postoperatively; T4, 48 h postoperatively.
