# Supplementary material for: Effects of preoperative aspirin on perioperative platelet activation and dysfunction in patients undergoing off-pump coronary artery bypass graft surgery: A prospective randomized study
Source: PLoS One. 2017 Jul 17;12(7):e0180466. doi: 10.1371/journal.pone.0180466 (PMC5513419; doi:10.1371/journal.pone.0180466)
Supplement: S2 Table — Results are shown as mean ± SD. Data were analyzed using a linear mixed-effect model for repeated measures with Bonferroni adjustment. No significant difference was observed between groups. CT, Clotting time; A10, amplitude of clot firmness 10 min after CT; CFT, clot formation time; MCF, maximum clot firmness. (DOCX) [file pone.0180466.s004.docx]

Table S2. ROTEM^®^ profile.

|  | Aspirin continuation (n = 24) | | | | Aspirin discontinuation (n = 24) | | | |
| --- | --- | --- | --- | --- | --- | --- | --- | --- |
|  | T1 | T2 | T3 | T4 | T1 | T2 | T3 | T4 |
| EXTEM-CT | 73 ± 53 | 65 ±13 | 65 ± 8 | 63 ± 14 | 70 ± 11 | 76 ± 39 | 68 ± 18 | 65 ± 14 |
| INTEM-CT | 218 ± 155 | 214 ± 41 | 173 ± 31 | 184 ± 36 | 194 ± 39 | 247 ± 82 | 197 ± 32 | 201 ± 79 |
| HEPTEM-CT | - | 218 ± 51 | 190 ± 32 | 191 ± 35 | - | 290 ± 139 | 203 ± 51 | 191 ± 46 |
| EXTEM-A10 | 56 ± 8 | 46 ± 7 | 51 ± 5 | 56 ± 7 | 57 ± 6 | 49 ± 9 | 52 ± 10 | 54 ± 12 |
| FIBTEM-A10 | 18 ± 6 | 12 ± 5 | 16 ± 5 | 24 ± 5 | 19 ± 6 | 14 ± 5 | 17 ± 5 | 22 ± 7 |
| EXTEM-CFT | 99 ± 51 | 140 ± 47 | 118 ± 25 | 97 ± 28 | 81 ± 20 | 127 ± 72 | 126 ± 91 | 97 ± 44 |
| INTEM-CFT | 88 ± 78 | 124 ± 53 | 92 ± 21 | 84 ± 24 | 66 ± 15 | 128 ± 111 | 99 ± 61 | 106 ± 93 |
| EXTEM-MCF | 64 ± 6 | 55 ± 7 | 60 ± 4 | 64 ± 6 | 67 ± 6 | 57 ± 8 | 60 ± 10 | 64 ± 10 |
| INTEM-MCF | 61 ± 7 | 54 ± 7 | 60 ± 4 | 62 ± 4 | 64 ± 5 | 55 ± 7 | 59 ± 8 | 64 ± 6 |

Results are shown as mean ± SD.

Data were analyzed using a linear mixed-effect model for repeated measures with Bonferroni adjustment.

No significant difference was observed between groups.

CT, Clotting time; A10, amplitude of clot firmness 10 min after CT; CFT, clot formation time; MCF, maximum clot firmness.
