# Supplementary material for: Effects of preoperative aspirin on perioperative platelet activation and dysfunction in patients undergoing off-pump coronary artery bypass graft surgery: A prospective randomized study
Source: PLoS One. 2017 Jul 17;12(7):e0180466. doi: 10.1371/journal.pone.0180466 (PMC5513419; doi:10.1371/journal.pone.0180466)
Supplement: S2 File — (DOCX) [file pone.0180466.s006.docx]

**연 구 계 획 서**

| 심폐우회술 없이 시행한 관상동맥 우회술에서 수술 전 저용량 아스피린의 사용이 혈소판 mRNA 발현에 미치는 효과 |
| --- |

IRB 통과시점 – IRB 통과 후 12개월

2014. 11.

서울대학교 의과대학 마취통증의학교실

부교수 전 윤 석

**Study outline**

| 연 구 제 목 | 심폐우회술 없이 시행한 관상동맥 우회술에서 수술 전 저용량 아스피린의 사용이 혈소판 mRNA 발현에 미치는 효과 |
| --- | --- |
| 연 구 목 적 | 혈소판 응집은 관상동맥 우회술 후 혈전 형성에 중요한 부분을 차지한다. 관상동맥 우회술 후 이를 조절하는 혈소판 mRNA와 단백질의 상승이 밝혀진 바, 본 연구에서는 심폐우회술 없이 시행한 관상동맥우회술(OPCAB) 전 저용량의 aspirin을 지속 복용한 환자와 복용하지 않은 환자에서 수술 후 3일째의 혈소판 mRNA 발현을 관찰하고자 한다. |
| 연 구 기 관 | 서울대학교병원 마취통증의학과 |
| 연 구 책 임 자 | 전 윤 석 |
| 연 구 대 상 | 정규로 OPCAB이 예정된 관상동맥질환 환자 |
| 연 구 기 간 | IRB 통과시점 – IRB 통과 후 12개월 |
| 연 구 방 법 | 정규 OPCAB 예정인 48명의 환자를 대상으로 수술 전 aspirin 100mg을 지속 투여한 군(n=24)과 수술 전 4일간 aspirin을 투여하지 않는 군(n=24)으로 무작위 배정한다. 주어진 시점에 혈소판 활성도 및 반응성 검사와 혈소판 mRNA 추출을 위한 혈액 샘플을 채취한다.  혈액 채취는 1) 피부 절개 전, 2) coronary artery bypass anastomosis가 끝나고 혈류 재개통 직후, 3) 수술 종료 시점, 4) 수술 종료 24시간 후, 5) 수술 종료 48시간 후, 6) 수술 종료 72시간 후, 7) 수술 종료 96시간 경과 시점에 시행한다. |
| 기대효과 및  예상결과 | 수술 전 저용량 aspirin 을 투여하지 않은 환자에게 있어서 OPCAB 후 혈소판 mRNA 발현 증가율이, 수술 전 aspirin을 지속 투여한 환자에 비교하여 높지 않을 것으로 예상된다. 본 연구 결과는 이제껏 명확히 표준화되어 있지 않았던 OPCAB 예정 환자에서의 항응고요법 정립의 근거가 될 수 있을 것으로 생각된다. |

**1. 연구 제목**

심폐우회술 없이 시행하는 관상동맥 우회술에서 수술 전 aspirin 지속 사용이 혈소판 mRNA 발현에 미치는 효과

**2. 실시기관명 및 주소**

서울특별시 종로구 대학로 101 서울대학교병원

**3. 연구 배경 및 목적**

**3.1 연구 배경**

관상동맥우회술 중 또는 수술 후 혈소판 응집, 혈소판 활성 및 반응성 증가, 기능 장애 등은 수술 중 혈전 형성이나 출혈 경향 등의 심각한 합병증을 가져 올 수 있다. 그 중에서도 동맥 혈전은 치명적인 결과를 초래할 수 있기 때문에 이러한 혈전 형성을 막기 위해서 수술 중과 수술 후 혈소판 응집을 막고, 혈액 응고성을 증가시키지 않는 요인들에 대해 많은 연구들이 진행되어 왔다.

특히 심폐기를 사용하는 관상동맥우회술에서의 혈소판 기능 이상은 발표된 모든 연구에서 수술 전과 비교하여 수술 후 유의하게 증가되었다. 이에 따라 심폐기를 사용하지 않는 관상동맥우회술을 시행하면, 심폐기에 의한 혈소판 기능 이상에 의한 부작용은 줄어들 것이라는 가설이 세워졌다. Bendar 등이 On pump CABG 환자와 OPCAB 환자에서 p-selectin의 증가율을 평가하여 수술 시점에 따른 혈소판 기능 변화를 유추하였고, OPCAB에서 수술 후 2일째에 혈소판 활성화 marker인 p-selectin의 증가율이 유의하게 가장 높은 것으로 발표하였다. Lo 등은 CPB 군에서 수술 직후 응고 활성화가 최대가 되는 것에 비교했을 때, OPcaB 군에서는 수술 후 20-96시간 사이에 응고 활성화가 최대가 된다고 보고하였다. Platelet activation과 관련된 연구는 p-selectin 뿐만 아니라, flow cytometry를 이용하여 혈소판 표면의 antigen을 측정함으로써 각기 다른 상황에 sensitive한 marker들로 연구될 수 있다. 가장 dominant 한 platelet activation marker로 P-selectin, PAC-1, CD 63 등이 있다. 이들을 이용하여 혈액관련 질환이나 항응고제제에 대한 반응 등을 관찰할 수 있다.

Aspirin은 심장 수술 환자들에게서 가장 많이 사용되는 항혈소판제이다. Aspirin은 혈소판 기능을 저하시키고 CPB 동안 출혈양을 증가시키는 것으로 알려져 있으나 많은 연구들에서 수술 전 저용량 aspirin 복용이 수술 후 출혈을 증가시킨다는 것에 대해 뚜렷한 근거를 제시하지는 못했다.

최근 aspirin에 대한 perioperative manage에 대한 견해는 매우 다양하다. The Society of Thoracic Surgeons에서는 수혈 관련 합병증을 줄이기 위해 정규 관상동맥 우회술 시행 전 3-5일간 aspirin 을 끊을 것을 추천하고 있다. 그러나 이에 반하여, American Society of Chest Physicians에서는 관상동맥 우회술을 시행할 때까지 aspirin을 지속 투여해야 할 것을 권유한다. The American College of cardiology and American Heart Association guideline에서는 관상동맥 우회술 전 7-10일간 aspirin 투약을 중단할 것을 추천하고 있다. Jacob 등은 관상동맥 우회술 전 4일 이내로 aspirin 투약을 중단하는 late discontinuation, 수술 전 7-10일간 aspirin 투약을 중단하는 early discontinuation 간에 수술 후 심혈관 합병증은 유의하게 차이 없이 비슷하였음을 발표하였다.

Bendar 등은 관상동맥 우회술 전 aspirin을 처치하지 않은 환자들에게서 수술 직후부터 aspirin 을 투약하고 시간에 따른 Thromboxane B_2_ 를 측정하였다. 이 연구에서 기준치에 비해 Thromboxane B_2_가 aspirin 투여 후 유의하게 감소한 것을 보여주었고, 이로 인해 aspirin 투여에 의한 혈소판 응집 감소를 보여 주어 관상동맥우회술 환자의 항혈소판제제 투여 전략에 도움을 주었다.

Reilly 등은 사람에게 있어 가장 강한 급성 염증 모델을 관상동맥우회술로 설명하였으며, 관상동맥우회술 후 3-6일 사이에 심근 경색 위험을 증가시키는 혈소판의 유전자가 대거 발현 됨을 최초로 연구, 발표하였다. 이들은 45개의 유전자를 검사하였고, 그 중 GPllb, Gpllla, COX-1 유전자 발현이 대상자 11명 전원에게서 기준치 대비 3배 이상으로 유의하게 증가하였다. 관상동맥 우회술로 인해 혈소판 응집에 주요 역할을 하는 mRNA와 단백질이 늘어나면, 이는 수술 후 심근 경색과 혈전 형성이 증가하는 요인이 될 수 있다.

기존 연구들에서는 수술 전 사용한 약제를 통제하지 못했다는 제한점이 있어서, 수술 전 저용량 aspirin 처치가 혈소판 응집에 관련하는 유전자 발현이나 혈소판 활성도에 미치는 영향을 구체적으로 보여주지는 못하였다. 항응고제와 항혈소판제의 혈소판 활성도에 대한 상호작용에 관한 연구 또한 많이 시행되었으나, 수술 전 투약에 대해서는 뚜렷한 방향을 제시하지 못하였고, 현재 관상동맥우회술 시행 이전 약제들의 사용은 센터마다 다른 실정이다. 따라서, 관상동맥질환 환자들의 혈전 형성을 줄이고자 하는 목적으로 사용되고 있는 수술 전 저용량 aspirin 사용이 혈소판 활성도와 혈소판 응집에 주요 역할을 하는 mRNA 및 단백질 발현에 어떠한 효과를 보이는지 약제를 통제한 연구가 필요하다.

**3.2 연구 목적 및 가설**

목적:

OPCAB 전 저용량(100mg)의 aspirin을 지속 복용한 환자와 수술 전 저용량 aspirin을 4일간 끊은 환자에서 수술 후 3일 째 혈소판 mRNA 발현을 관찰, 수술 전과 수술 후 3일째의 혈소판 mRNA의 증가율을 평가한다.

가설:

수술 전 4일간 저용량 aspirin을 투여하지 않은 환자에서 수술 후 3일 째 혈소판 활성 관련 mRNA의 증가율이, 수술 전 저용량 aspirin 지속 투여 군의 수술 후 3일째 혈소판 활성 관련 mRNA 증가율에 비해 높지 않다.

**4. 대상질환 및 대상자**

정규로 OPCAB이 예정된 관상동맥질환 환자

**5. 예상 연구기간**

IRB 통과시점 이후 12개월 (enroll이 지연될 경우, 더 길어질 수 있음.)

**6.** 연구대상자 선정 및 연구대상자 수

**6.1.** 연구대상자 의 선정 기준, 제외 기준

**선정 기준**

20세 이상의 성인 환자

정규로 최초 OPCAB이 예정된 관상동맥질환 환자

다음 제외 기준에 속하지 않는 환자로 연구에 동의한 경우

**제외 기준**

기존에 혈액 응고 또는 지혈 질환이 있는 경우

- 혈소판 수 < 100,000, PT INR >1.2, antithrombin lll <80% or > 120%, fibrinogen <2g/ L or >6g/L

응급 또는 재수술 (OPCAB)

판막과 좌심실 동맥류 수술이 동반된 경우

간 또는 신장 질환이 동반된 경우

MI를 겪고 12개월이 지나지 않은 경우

Unstable angina를 겪고 10일이 지나지 않은 경우

수술 30일 이전 이내 PCI를 받은 경우

Bare metal stent를 거치한 후 6주가 지나지 않은 경우

Drug eluting stent를 거치한 후 12개월이 지나지 않은 경우

수술 6개월 이내 뇌경색이 있었던 경우

Heparin induced thrombocytopenia가 있는 경우

Heparin resistance가 있는 경우

월경 중인 경우

연구에 동의하지 않는 경우

수술 전 Cardiopulmonary Bypass, Continuous veno-venous hemofiltration, Intraaortic balloon pump가 사용된 경우

Aspirin 이외 다른 항응고제를 사용하고 있으며, 수술 전 끊지 못하는 경우 (plavix, heparin, low molecular weight heparin)

**중도탈락 및 임상시험 중지 기준**

연구대상자의 동의 철회

수술 중 Cardiopulmonary Bypass, Continuous veno-venous hemofiltration, Intraaortic balloon pump가 사용된 경우

**6.2.** 연구대상자 수

Reilly 등의 연구에서 수술 전 aspirin을 투여하고 관상동맥우회술을 시행한 환자에서 수술 후 3-6일 사이에 심근 경색 위험을 증가시키는 혈소판의 유전자가 대거 발현됨이 발표되었다. GPllb, Gpllla, COX-1 유전자 발현이 대상자 11명 전원에게서 기준치 대비 3배 이상으로 유의하게 증가하였다. 심폐우회술 없이 시행한 관상동맥 우회술에 대해서는 GPllb, Gpllla, COX-1 유전자 발현에 관련된 기준 자료가 없어 pilot 연구로 군 당 20명씩을 산정하고, 이에 총 연구대상자 수 40명, 탈락률 20%를 고려하여 48명의 환자가 필요할 것으로 예상된다.

**7. 연구 일정**

IRB 통과 직후 –IRB 통과 후 12개월

IRB 통과 직후 1개월: 시험 준비

IRB 통과 1개월 후부터 9개월: 임상 시험

IRB 통과 10개월 후부터 2개월: 자료 정리 및 논문 작성

**8. 연구 방법**

**8.1 대상자 선정**

정규로 OPCAB을 받는 관상동맥질환 환자들 중 상기된 참여기준에 부합되고, 제외 기준에 속하지 않으며 연구대상자가 동의할 경우 대상자로 선정한다.

**8.2 연구대상자 동의 확보**

별첨된 설명문과 동의서에 따라 연구 담당자 또는 공동 연구자가 연구대상자에게 설명한 후 서면 동의를 받는다. 연구 설명 후 환자의 동의 취득 사이에 충분한 대기 시간을 가지도록 하며, 이 과정에서 설명문과 동의서 사본을 연구대상자에게 제공한다. 연구대상자의 참여의지에 영향을 줄 수 있는 새로운 정보가 수집되면, 즉시 연구대상자 또는 대리인에게 알려준다. 고위험군의 관상동맥우회술 예정 연구대상자, 또는 응급으로 수술을 받게 되는 경우가 아니라 정규로 예정된 수술을 받게 될 경우, 수술 시까지 3-5일간 aspirin을 중단함으로써 생기는 수술 전후 합병증은 극히 드문 것으로 알려져 있고, 비심장 수술의 경우에도 수술의 종류에 따라 수술 전 7일 가량 aspirin을 중단하기도 한다. 자발적인 의사로 참여하더라도 aspirin 투여 중지 군에서 유의한 흉통과 같은 중증도 이상의 이상반응이 생기면 즉시 연구를 중지하도록 한다.

**8.3 무작위 배정**

연구대상자는 다음과 같은 내용으로 1:1로 무작위 배정한다.

대조군: 수술 전 low dose aspirin (100mg/day)을 지속 복용 후 수술하는 군

시험군: 수술 4일 전 low dose aspirin (100mg/day)을 중단 후 수술하는 군

연구대상자 등록 전 본 연구에 관여하지 않는 의사가 대조군(A), 시험군(B)을 각각 1명씩 포함하는 크기가 2인 블록 (AA, AB, BA, BB)이 무작위로 섞인 블록 무작위 배정표를 난수표에 따라 미리 작성하고, 이 배정표에 따른 순서대로 연구대상자를 대조군이나 시험군에 배정하여 시험을 진행한다

**8.4. 임상 시험의 시행 계획**

정규 OPCAB 예정인 환자를 대상으로 수술 전까지 100mg/dl의 저용량 aspirin을 지속 투여하는 군(n=24)과 aspirin 투여를 4일간 중단하고 수술하는 군(n=24)으로 무작위 배정한다.

마취 유도 전 국소 마취 하 요골 동맥에 동맥 도관을 거치하고, 침습적 혈압 감시 및 BIS 모니터 하에 Midazolam 0.15~0.3mg/kg, sufentanil 1~2mcg/kg, vecuronium으로 전신 마취를 시행한다. 수술 중 마취는 전정맥마취로 유지한다. OPCAB 수술 중 목표 ACT를 250-350초로 하여 Y graft 제작 전 비분획형 heparin 1.5mg/kg를 투여한 후 다음과 같이 정해진 시점에 혈소판 활성도 및 반응성 검사를 시행한다. 아래 각 시점마다 15ml의 혈액 샘플이 필요하며, 시점 1), 6)에서는 혈소판 mRNA 검사를 위해 30 ml씩의 혈액 채취가 추가로 필요하다.

1) 피부 절개 전,

2) coronary artery bypass anastomosis가 끝나고 혈류 재개통 직후,

3) 수술 종료 시점,

4) 수술 종료 24시간 후,

5) 수술 종료 48시간 후

6) 수술 종료 72시간 후

7) 수술 종료 96시간 후

**8.5. 관찰 항목, 평가 변수**

9.4에 기술한 시점에 혈액 응고 검사 및 혈소판 활성도, 반응성 검사를 시행한다.

- Platelet count

- Conventional coagulation tests (CCT : INR, aPTT, fibrinogen)

- Activated clotting time (ACT)

- Capillary closure time (PFA-100/EPI, PFA-100/ADP)

- D-dimer

- von Willebrand factor

- Rotational thromboelastometry (ROTEM)

- Platelet function (Verify/Now accumetrics)

- Platelet aggregation (multiplate , inducer : collagen, ADP, TRAP-6, arachidonic acid)

- Annexin V (flow cytometry)

- platelet P-selectin (flow cytometry)

- platelet CD41(flow cytometry)

- PAC-1 (flow cytometry)

- platelet CD63( flow cytometry)

- Thromboxan B_2_

위 시점 중 1), 6)에 다음 검사를 시행한다.

- GPllb, GPllla, COX-1 mRNA (RT-PCR)

**추가관찰 항목**

- 수술 중 출혈량

- 수술 후 흉관에 배액된 출혈량

- 수술 후 경과 (MACE : MI, stroke, coma, arrest), renal failure, readmission

**일차평가변수**

수술 전과 비교하여, 수술 후 3일째 RT-PCR을 이용하여 측정한 혈소판의 GPllb mRNA 발현의 증가율 (수술 전 baseline으로 측정한 혈소판 GPllb mRNA에 비하여 수술 후 3일째 측정한 혈소판 GPllb mRNA가 증가한 정도 - %)

**8.6 판정 기준 및 통계 분석**

통계적 분석은 intention-to-treat로 이루어지며, SPSS 21.0 for windows (SPSS, Chicago, IL, USA)를 이용한다.

Aspirin 투여 군과 투여하지 않은 군의 임상 변수들의 군 간 차이는 정규 분포를 따르는 경우 student t-test 또는 Chi square test를 시행하며, 정규 분포를 따르지 않는 경우 Mann-Whitney test 또는 Fisher’s exact test를 사용한다.

일차 평가 변수인 수술 후 3일째의 혈소판 GPllb mRNA 발현의 증가율은 독립 t-test를 사용한다. 기준점에서부터 혈액 채취 시점, 군 간(preoperative aspirin vs control), 혈소판 mRNA 발현을 포함한 약제의 상호 작용 효과의 증감율을 평가할 때 Generalized estimating equation / general linear model을사용하며, p값이 0.05 미만일 때 통계적으로 유의한 것으로 평가한다. 시점, 군, 상호 작용 효과가 통계적으로 유의할 때, 시점 간 자료의 비교는 RM ANOVA with Bonferroni correction을 이용한다.

수술 중 출혈량, 수술 후 흉관에 배액된 출혈량의 군간 비교는 독립 t-test를 이용한다.

**9. 참고문헌**

1. Bernard Lo, Rob Fijnheer, Domenico Castigliego, Cornelius Borst, Cor J. Kalkman, Arno P. Nierich, Activation of hemostasis after coronary artery bypass grafting with or without cardiopulmonary bypass. Anesth Analg 2004; 99: 634-40

2. Alessandro Parolari, Luciana Mussoni, Marta Frigerio, Moreno Naliato et al., The role of tissue factor and P-selectin in the procoagulant response that occurs in the first month after on-pump and off-pump coronary artery bypass grafting. The journal of thoracic and cardiovascular surgery 2005; 130(6): 1561-1566

3. Raymond Cartier, Current trends and technique in OPCAB surgery. J Card Surg 2003; 18: 32-46

4. Frantisek Bendar, Pavel Osmancik, Tomas Vanek, Heidi Mocikova, Martin Jares, Zbynek Straka, Petr Widmsky , Platelet activity and aspirin efficacy after off-pump compared with on-pump coronary artery bypass surgery: results from the prospective randomized trial PRAGUE 11-Coronary Artery Bypass and Reactivity of Thrombocytes (CABARET). The journal of thoracic and cardiovascular surgery 2008; 136(4): 1054-1060

5. Robert Poston, Junyan Gu, James Brown, James Gammie, Charles White, Jeffrey Manchio, Richard N. Pierson et al., Hypercoagulability affecting early vein graft patency dose not exist after off-pump coronary artery bypass. J of cardiothoracic and vascular anesthesia 2005; 19(1): 11-18

6. Brian R. Untch, Walter P. Jeske, Jeffrey Schwartz, Sally Botkin, Margaret Prechel, Jeanine M. Walenga and Mamdouh Bankhos, Inflammatory and hemostatic activation in patients undergoing off-pump coronary artery bypass grafting. Clin Appl Thromb Hemost 2008; 14(2): 141-8

7. Andrea Ballotta, Hisham Z. Saleh, Hisham W. El Baghdady, Magdi Gomaa et al., Comparison of early platelet activation in patients undergoing on-pump versus off-pump coronary artery bypass surgery. The journal of thoracic and cardiovascular surgery 2007; 134(1): 132-138

8. Grzegorz Suwalski, Piotr Suwalski, Krzysztof J. Filipiak, Marek Postula, Franciszek Majstrak et al., The effect of off-pump coronary artery bypass grafting on platelet activation in patients on aspirin therapy until surgery day. European journal of cardiothoracic surgery surgery 2008; 34: 365-369

9. F. Bendar, T. Tencer, P. Plasil, Z. Paluchet al., Evaluation of aspirin’s effect on platelet function early after coronary artery bypass grafting. J of Cardiothoracic and vascular anesthesia 2012; 26(4); 575-580

10. S. J. Reilly, J. Liska, M. Ekstrom et al., Coronary artery bypass graft surgery-upregulates genes involved in platelet aggregation. J of thrombosis and haemastasis 2012; 10: 557-63

11. Eagle KA, Guyton RA et al., ACC/AHA 2004 guidelines update for coronary artery bypass graft surgery; a report of the American Collage of Cardiology/American Heart Association Task Force on practice Guidelines

12. P. S. Myles,Stopping aspirin before coronary artery surgery. Circulation 2011; 123: 571-573

13. Mirian Jacob et al., Effect of chronic preoperative aspirin discontinuation on morbidity and mortality in coronary artery bypass surgery. Circulation 2011; 123: 577-583

14. Jeroen F. van Velzen et al., Multicolor flow cytometry for evaluation of platelet surface antigens and activation markers. Thrombosis research 2012; 130(1):92-8
