# Supplementary material for: Effects of preoperative aspirin on perioperative platelet activation and dysfunction in patients undergoing off-pump coronary artery bypass graft surgery: A prospective randomized study
Source: PLoS One. 2017 Jul 17;12(7):e0180466. doi: 10.1371/journal.pone.0180466 (PMC5513419; doi:10.1371/journal.pone.0180466)
Supplement: S3 File — (DOCX) [file pone.0180466.s007.docx]

**Research Proposal**

Title : Effects of low dose aspirin pre-treatment on platelet mRNA expression in patients undergoing off-pump coronary artery bypass graft

**Background**

There are some problems such as platelets aggregation, increase of platelets activation, and functional disorder leading to thrombosis formation or bleeding during intraoperative and postoperative periods in coronary artery bypass surgeries. Because arterial thrombosis formation can cause serious outcome, some studies investigated various factors to prevent platelet aggregation and to inhibit increase of coagulation of blood.

Previous studies suggested that abnormality of platelet function increased at postoperative period compared to preoperative period in cases which CPB was used in the operation. Therefore, there was a hypothesis that abnormality of platelet function caused by CPB would lesser develop in off-pump CABG compared to CABG. Bendar et al. presented that changes of platelet function by evaluating increase of p-selectin in patients who received OPCAB or CABG. They reported that increase of p-selectin as a platelet activation marker was highest at 2 days after operation in patients who received OPCAB. Lo et al. reported that platelet activation in terms of coagulation was maximized at immediate postoperative period in CABG, and between 20 – 96 hours after operation in OPCAB. There are some methodologies in evaluating platelet activation, that is, not only p-selectin but also flow cytometry by which antigen on the surface of platelet can be estimated. The most dominant platelet activation markers are p-selectin, PAC-1, CD 63. They can be used to evaluate blood disorders or responses to anticoagulants.

Aspirin was a most used anticoagulant in patients who received cardiac surgeries. It is well known that aspirin decrease platelet function and increase bleeding tendency in CPB. However, there has been any strong evidence supporting that aspirin administration lead to increase of postoperative bleeding.

There are various opinions on the perioperative use of aspirin for OPCAB or CABG. The Society of Thoracic Surgeons recommends stop of aspirin use 3 days before operation to minimize complications concerned with blood transfusion. American Society of Chest Physicians recommended continuous use of aspirin even at operation day. The American College of Cardiology and American Heart Association recommended stop of aspirin use 7-10 days before OPCAB or CABG. Jacob et al. reported that there was no difference between late discontinuation (stop 4 days before surgery) and early discontinuation (stop 7-10 days before surgery) on the perioperative complications.

Bednar et al. investigated the effect of aspirin administration at immediate postoperative periods on the level of Thromboxane B2 in patient who did not receive preoperative aspirin for CABG. The study reported that thromboxane B2 decreased significantly by postoperative aspirin administration. This study helped many physicians make a strategy of anticoagulants use in CABG.

Rally et al. presented that the model of most strong acute inflammation in human could be explained with coronary artery bypass surgery. They showed outbreak of expression of a gene influencing platelet at 3-6 days after surgery which could increase risk of myocardiac infarction. They evaluated 45 genes. GpIIb, GpIIIa, and Cox-1 gene expression did significantly increase by 3 times compared with baseline in all 11 subjects in the study. If any mRNA and protein which have a great role in platelet aggregation would increase due to CABG, this could be an important factor cause increase of thrombosis.

Because previous studies did not control preoperative use of anticoagulants, they could not reveal the effect of preoperative aspirin use on gene expression which influence platelet aggregation or platelet activation. There were numerous studies about co-effect of antiplatelet agents and anticoagulants on the activity of platelet. However, there has been no consensus about preoperative use of them. Therefore, strategies of perioperative use of them are various according to centers. Therefore, a study on the effect of preoperative low dose aspirin use on the mRNA which have a great role on platelet activation and platelet aggregation ant protein expression by it should be needed.

**Purpose of study and Hypothesis**

**Purpose**

The purpose of this study is to evaluate the difference between mRNA estimated at preoperative periods and 3 days after operation in patients who received continuously low dose (100 mg) of aspirin until operating day and in patients who stopped aspirin 4 days before surgery. .

**Hypothesis**

Our hypothesis is that increase of mRNA at 3 days after surgery compared with preoperative value in patients who stopped aspirin 4 days before surgery will not be higher than it in patients who continue aspirin until operating day.

**Subject**

Patients receiving an elective OPCAB

**Expected study period**

12 months from IRB’s approval

**Inclusion criteria**

Adult patients more than 20 years old

Patient receiving an elective OPCAB, which is a first cardiac surgery to the patient

**Exclusion criteria**

Previous hematologic disease

Disorders of coagulation

Preoperative Plt. count < 100,000.

Preoperative PT INR > 1.2

Preoperative antithrombin III < 80% or 120%

Fibrinogen < 2g/L or 6g/L

Emergent operation

Reoperation of OPCAB

Combined operation with valvular surgery or aortic aneurysmal surgery

Hepatic disease

Renal disease

Recent MI within 12 months

Recent unstable angina within 10 days

Recent PCI history within 30 days

Implantation of Bare metal stent within 6 weeks

Implantation of drug eluting stent within 12 months

Recent cerebral infarct within 6 months

Heparin induced thrombocytopenia

Heparin resistance

Menstruation

No approval of study

Preoperative use of cardiopulmonary bypass, continuous veno-venous hemofiltration, or intraarotic balloon pump

Continuous use of other anticoagulants except aspirin such as plavix, heparin, low molecular weight heparin until operating day

**Criteria of drop-out**

Declaration of approval

Intraoperative use of cardiopulmonary bypass, continuous veno-venous hemofiltration, or intra-aortic balloon pump

**Number of subjects**

Reilly reported that outbreak of gene concerned with platelet which could increase risk of MI occurred at 3-6 days after operation in patients who received continuously aspirin until operating day before coronary artery bypass surgery. In the study, GPIIb, GpIIIa, COX-1 gene expression increased by 3 times in all of 11 patients compared to baseline. For OPCAB, there was no data on GpIIb, GpIIIa, COX-1 gene expression. Therefore, 20 subjects are needed for each group, that is, total of 40 subjects are needed for this study. Considering the loss rate of 20%, total of 48 subjects will be needed.

**Randomization**

All subjects will be randomly assigned with 1:1 ratio to two groups as following:

- Control group: patients receiving low dose aspirin (100 mg/day) until operating day
- Study group: patients stop aspirin (100 mg/day) 4 days before surgery

Random sequence will be made by a study nurse with size 2 blocks (AA, AB, BA, BB) including letter of ‘A’ (meaning aspirin continuation group) and ‘B’ (meaning aspirin discontinuation group). And then each generated letter will be concealed in an envelope in the order of generation. Enrolled patients will be allocated to their groups depending on the letter (A or B) inside the concealed envelopes that will be opened by an anesthesiologist who was unaware of the study.

**Study protocol**

Assigned ward nurses will deliver drugs to all patients during the perioperative period.

In the aspirin continuation group (n=24), aspirin (100 mg; aspirin protect®, Bayer AG, Leverkusen, Germany) will be administered every morning until the operative day.

In the aspirin discontinuation group (n=24), aspirin will be stopped 4 days before the operative day.

Other anticoagulants, such as clopidogrel or warfarin will be stopped at least 5 days before surgery in all patients.

Anesthetic and surgical techniques will be standardized during the trial. A single anesthesiologist and a single surgeon, not aware of the group assignment, will perform OPCAB in all patients. All patients will arrive in the operating room without premedication. A catheter will be inserted at radial artery after local anesthesia for continuous arterial blood pressure monitoring. And standard monitoring including ECG, NIBP, SpO_2_ and BIS monitoring. Anesthesia will be induced with 0.15 mg/kg midazolam, 1-2 μg/kg sufentanil and vecuronium. After endotracheal intubation, mechanical ventilation will be applied to maintain an end-tidal carbon dioxide tension of 35-40 mmHg. Anesthesia will be provided with target-controlled infusion of propofol and remifentanil.

Target activated clotting time (ACT) will be 250-350 s.

1.5 mg/kg unfractionated heparin will be administrated before making of Y-graft.

At following time points, blood will be sampled for evaluation of platelet activation and responsibility.

- T1. Before skin incision
- T2. After reperfusion of graft
- T3. End of surgery
- T4. 24 h postoperatively
- T5. 48 h postoperatively
- T6. 72 h postoperatively
- T7. 96 h postoperatively

**Laboratory evaluation lists**

At the time points (T1, T2, T3, T4, T5, T6, T7)

- Platelet count

- Conventional coagulation tests (CCT : INR, aPTT, fibrinogen)

- Activated clotting time (ACT)

- Capillary closure time (PFA-100/EPI, PFA-100/ADP)

- D-dimer

- von Willebrand factor

- Rotational thromboelastometry (ROTEM)

- Platelet function (Verify/Now accumetrics)

- Platelet aggregation (multiplate , inducer : collagen, ADP, TRAP-6, arachidonic acid)

- Annexin V (flow cytometry)

- platelet P-selectin (flow cytometry)

- platelet CD41(flow cytometry)

- PAC-1 (flow cytometry)

- platelet CD63( flow cytometry)

- Thromboxan B2

At the time points (T1, T6)

- GPllb, GPllla, COX-1 mRNA (RT-PCR)

**Clinical evaluation lists**

- volume of intraoperative bleeding

- volume of postoperative bleeding

- postoperative clinical outcomes : MACE (MI, stroke, coma, arrest), renal failure, re-admission

Primary Outcomes

Increase (%) of GPIIb mRNA at 96 h postoperative time point (T6) compared to baseline (before initiation of surgery, T1)

**Statistical analyses**

Statistical analyses will be performed with intention-to-treat.

If data will be normal distributed, we will use student t-test or Chi square test. Otherwise, Mann-Whitney test or Fisher’s exact test will be used.

For the primary outcome, increase of GPIIb mRNA, independent t-test will be used.

For the evaluation discrepancy of platelet mRNA expression by aspirin effect between two groups at each time points, we will use the generalized estimating equation/general linear model. P-value < 0.05 will be considered to be significant.

Repeated-measures analysis and Bonferroni correction will be used for adequate analysis between groups.

Data will be presented as mean ± Standard deviation, median [interquartile range], and number of patients (%). All statistical analyses will be performed with IBM SPSS Statistics software (version 21, SPSS Inc., IBM Corporation, USA).

References

1. Bernard Lo, Rob Fijnheer, Domenico Castigliego, Cornelius Borst, Cor J. Kalkman, Arno P. Nierich, Activation of hemostasis after coronary artery bypass grafting with or without cardiopulmonary bypass. Anesth Analg 2004; 99: 634-40

2. Alessandro Parolari, Luciana Mussoni, Marta Frigerio, Moreno Naliato et al., The role of tissue factor and P-selectin in the procoagulant response that occurs in the first month after on-pump and off-pump coronary artery bypass grafting. The journal of thoracic and cardiovascular surgery 2005; 130(6): 1561-1566

3. Raymond Cartier, Current trends and technique in OPCAB surgery. J Card Surg 2003; 18: 32-46

4. Frantisek Bendar, Pavel Osmancik, Tomas Vanek, Heidi Mocikova, Martin Jares, Zbynek Straka, Petr Widmsky , Platelet activity and aspirin efficacy after off-pump compared with on-pump coronary artery bypass surgery: results from the prospective randomized trial PRAGUE 11-Coronary Artery Bypass and Reactivity of Thrombocytes (CABARET). The journal of thoracic and cardiovascular surgery 2008; 136(4): 1054-1060

5. Robert Poston, Junyan Gu, James Brown, James Gammie, Charles White, Jeffrey Manchio, Richard N. Pierson et al., Hypercoagulability affecting early vein graft patency dose not exist after off-pump coronary artery bypass. J of cardiothoracic and vascular anesthesia 2005; 19(1): 11-18

6. Brian R. Untch, Walter P. Jeske, Jeffrey Schwartz, Sally Botkin, Margaret Prechel, Jeanine M. Walenga and Mamdouh Bankhos, Inflammatory and hemostatic activation in patients undergoing off-pump coronary artery bypass grafting. Clin Appl Thromb Hemost 2008; 14(2): 141-8

7. Andrea Ballotta, Hisham Z. Saleh, Hisham W. El Baghdady, Magdi Gomaa et al., Comparison of early platelet activation in patients undergoing on-pump versus off-pump coronary artery bypass surgery. The journal of thoracic and cardiovascular surgery 2007; 134(1): 132-138

8. Grzegorz Suwalski, Piotr Suwalski, Krzysztof J. Filipiak, Marek Postula, Franciszek Majstrak et al., The effect of off-pump coronary artery bypass grafting on platelet activation in patients on aspirin therapy until surgery day. European journal of cardiothoracic surgery surgery 2008; 34: 365-369

9. F. Bendar, T. Tencer, P. Plasil, Z. Paluchet al., Evaluation of aspirin’s effect on platelet function early after coronary artery bypass grafting. J of Cardiothoracic and vascular anesthesia 2012; 26(4); 575-580

10. S. J. Reilly, J. Liska, M. Ekstrom et al., Coronary artery bypass graft surgery-upregulates genes involved in platelet aggregation. J of thrombosis and haemastasis 2012; 10: 557-63

11. Eagle KA, Guyton RA et al., ACC/AHA 2004 guidelines update for coronary artery bypass graft surgery; a report of the American Collage of Cardiology/American Heart Association Task Force on practice Guidelines

12. P. S. Myles,Stopping aspirin before coronary artery surgery. Circulation 2011; 123: 571-573

13. Mirian Jacob et al., Effect of chronic preoperative aspirin discontinuation on morbidity and mortality in coronary artery bypass surgery. Circulation 2011; 123: 577-583

14. Jeroen F. van Velzen et al., Multicolor flow cytometry for evaluation of platelet surface antigens and activation markers. Thrombosis research 2012; 130(1):92-8
