# Supplementary material for: Effects of preoperative aspirin on perioperative platelet activation and dysfunction in patients undergoing off-pump coronary artery bypass graft surgery: A prospective randomized study
Source: PLoS One. 2017 Jul 17;12(7):e0180466. doi: 10.1371/journal.pone.0180466 (PMC5513419; doi:10.1371/journal.pone.0180466)
Supplement: S4 File — (PDF) [file pone.0180466.s008.pdf]

SNUCM/SNUH IRB

Seoul National University College of Medicine/Seoul National University Hospital
Institutional Review Board

Tel :82-2-2072-0694
FAX:82-2-2072-0368 101 Daehak-ro, Jongno-gu, Seoul, 110-744, Korea

IRB Decision Notification Document

THE FOLLOWING WERE APPORVED:

IRBNO : H-1310-046-526

RESEARCH TITLE : Effects of low dose aspirin pre-treatment on platelet mRNA expression in patients undergoing off-pump coronary artery bypass graft

INVESTIGATOR : YUN SEOK JEON

SPONSOR :

REVIEW LIST:
1. Protocol
2. Information Sheet and Informed Consent
3. Case Report Form
4. Indemnification Provisions for Subjects
5. Principal Investigator's Recent Curriculum Vitae

Review Comment:
- According to IRB approval criteria, the IRB approves the research.

ALL CONDITIONS OF APPROVAL PREVIOUSLY ESTABLISHED BY SNUCM/SNUH IRB
FOR THIS RESEARCH PROJECT CONTINUE TO APPLY.

CONTINUING REVIEW REPORT INTERVAL: (6) Month

IF YOU HAVE ANY QUESTIONS, CONTACT SNUCM/SNUH IRB (Tel: 82-2-2072-0694)

This is to certify that the information contained herein is true and correct as reflected in the records of the SNUCM/SNUH IRB. **We certify that SNUCM/SNUH IRB is in full compliance with Good Clinical Practice as defined under the Korean Ministry of Food and Drug Safety (MFDS) regulations and the International Conference on Harmonisation (ICH) guidelines.**

Byeongfoo Park 2013-12-10

Chairperson **Date**

All investigators performing SNUBH IRB approved projects must comply with the followings:

1. Enrollment of participated subjects before the IRB approval of protocol/protocol amendment is forbidden.

2. To conduct the study according to the approved protocol. To conduct the study differently from the original protocol is forbidden.

3. To use the approved Informed Consent Form.

4. The informed consent process shall be conducted based on sufficient explanation under no coercion or unfair influence, and a potential subject shall be provided with sufficient opportunity to consider the study participation.

5. Except for the unavoidable cases to protect subjects during the study conduct, any amendment of the study shall be implemented after getting the prior approval of the Board, and any amendment taken in an emergency situation for protection of subjects shall be immediately reported to the Board.

6. In case the study should be conducted differently from the original protocol since the immediate risk factor occurring to subjects should be eliminated, the amendment item that may increase risk factors occurring to subjects or have serious effects of the study conduct, items on the unexpected serious adverse drug reaction, or items on new information that may have negative effects on subjects' safety or study conduct shall be promptly reported to the Board.

7. The subject recruitment advertisement approved by the Board shall be used.

8. The Board approval period may not exceed one year. In case of intending to continue the study for more than one year, you are required to submit an annual continuation report.

9. In case the IRB review decision is not an 'Approval', written response for IRB decision result shall be submitted within six months since the IRB review date.

10. In the case of a decision by the Board to disapprove, you may have the opportunity to submit an appeal in writing. However, you should not file an appeal 2 times in a row with the same reason.

11. When completing the research, Study completion report and Study result report shall be submitted.

12. You shall comply with Bioethics and Safety Act, Pharmaceutical Affairs Act/Medical Device Affairs Act as defined under the Ministry of Food and Drug Safety (MFDS) regulations, the International Conference on Harmonization (ICH) guidelines and the Declaration of Helsinki.

13. According to the Declaration of Helsinki, all clinical studies shall be disclosed in the database that allows public access(primary registry) prior to the first subject enrollment; for example, you may use http://register.clinicaltrials.gov. For details, please refer to the IRB website.

14. The internal audit or inspection from the regulatory agency for the approved study could be conducted. Investigator shall cooperate in helping this to carry out, when requested for reading of study document (including electronic document) by internal auditor or monitor from sponsor, or inspector from regulatory agency.

This is to certify that the information contained herein is true and correct as reflected in the records of the SNUCM/SNUH IRB. **We certify that SNUCM/SNUH IRB is in full compliance with Good Clinical Practice as defined under the Korean Ministry of Food and Drug Safety (MFDS) regulations and the International Conference on Harmonisation (ICH) guidelines.**
